# 

**Published:** 1904-07-16

**Authors:** 


### NOTICE TO CORRESPONDENTS.

All MSS., Letters, Books for Review, and other matters intended for the Editor should be addressed The Editor, "The Hospital," The Hospital Buildings, 28 & 29 Southampton Street, Strand, W.C.

The Editor cannot undertake to return rejected MS., even when accompanied by stamped directed envelope.

### Notice to Advertisers and Subscribers.

All Advertisements, Orders for Copies of The Hospital, and Business Communications should be addressed to **THE MANAGER (Not to the Editor)**, The Hospital, 28 & 29 Southampton Street, Strand, London, W.C.

The Cost of Subscription, post free, is as follows:—Per week, 3½d.; per quarter, 3s. 3d.; per half-year, 6s. 6d.; per annum, 13s. Foreign and Colonial:—Per annum, 19s. 6d., payable, in all cases, in advance.

**NOTICE.—Vol. XXXV. of "The Hospital" (October 1903 to March 1904), in handsome cloth binding, is now on sale at the office of this Journal, price 10s. 6d. Cases for binding the Numbers contained in this Volume 2s. Index to Vol. XXXV., 3½d., post free.**

**The Monthly part for July is now ready. Price 1s., by post, 1s. 3d.**

### MEDICAL APPOINTMENTS.

**E. D. Aubin**, M.R.C.S., L.R.C.P., Health Officer for the Port of Thames, New Zealand. **W. H. Bateman**, M.B., Ch.B.Vict., Medical Officer of Health to the Norden Urban District Council. **F. G. Bushnell**, M.D., B.S.Lond., P.H.D.Camb., Pathologist to the proposed Ralli Memorial Laboratories, Sussex County Hospital, Brighton. **Frederick Chapple**, M.B., Ch.B., Medical Superintendent to the Adelaide Hospital. **W. H. Clayton-Greene**, B.A., M.B., B.C.Cantab., F.R.C.S.Eng., Supernumerary Surgeon in charge of Out-patients to St. Mary's Hospital, Paddington. **F. W. E. Courtenay**, L.R.C.P., L.R.C.S., etc., Medical Referee to Empire Guarantee and Insurance Corporation, Limited. **A. Cruickshank**, M.B., C.M.Aberd., Certifying Factory Surgeon for the District of Stonehaven. **Robert Dey**, M.B.Syd., Government Medical Officer and Vaccinator at Bourke, N.S.W. **A. T. Emmerson**, M.D.Toronto, Clinical Assistant to the Chelsea Hospital for Women. **James A. Gibb**, M.B., Ch.B.Aberd., Assistant Medical Officer to the Dorset County Asylum. **R. R. Hatherall**, M.R.C.S., District Medical Officer of the Abingdon Union. **Joseph F. S. Hay**, M.B., C.M.Aberd., Inspector of Lunatic Asylums, Hospitals, and Licensed Houses in the Colony of New Zealand. **A. E. Jerman**, M.B.Lond., M.R.C.S.Eng., Medical Officer of Health to the Erith Urban District. **H. Mulrea Johnston**, B.A., M.B., B.Ch., Chief Demonstrator of Anatomy in Trinity College, Dublin. **W. R. Kelly**, M.B., B.S.Melb., Medical Officer at Croydon, Queensland. **J. H. Lilley**, M.D.Camb., M.R.C.S., Medical Officer of the Children's Homes of the Hereford Union. **G. E. Lockyer**, M.R.C.S., L.R.C.P., District and Workhouse Medical Officer of the Amesbury Union. **Frederick S. Palmer** M.D., M.R.C.P., Assistant Physician to the West End Hospital for Diseases of the Nervous System, Welbeck Street, W. **J. P. Philip**, M.D.Aberd., D.P.H., Medical Officer of Health to the Morpeth Rural District Council. **W. H. Read**, M.B., Ch.M.Syd., Honorary Assistant Skiagraphist, Sydney Hospital, N.S.W. **A. Rotherham**, M.B., B.C.Camb., Medical Superintendent of Darenth Asylum. **Robert James Rowlette**, B.A., M.D.Dub., Anæsthetist to the Incorporated Dental Hospital of Ireland. **Wilfrid Sass**, M.R.C.S.Eng., L.R.C.P.Lond., Assistant Anæsthetist to the Cancer Hospital, Brompton, S.W. **W. Stephen**, B.A., M.B.Toronto, Clinical Assistant, Chelsea Hospital for Women. **W. B. Vaile**, M.R.C.S., L.R.C.P., Resident Assistant Medical Officer, West Ham Union Workhouse. **S. G. Vinter**, M.R.C.S., L.R.C.P., D.P.H., Medical Officer of Health, Torpoint Urban District. **R. J. Waugh**, M.R.C.S., L.R.C.P., Third Assistant Medical Officer, Bethnal Green Infirmary. **D. J. Williams**, M.R.C.S.Eng., L.R.C.P.Lond., Medical Superintendent of the Lunatic Asylum, Jamaica.

**"The Hospital" Institutional Library.**

**"Hospitals and Asylums of the World." 4 Vols., with a Portfolio of Plans, complete £12 12s.**

**"Burdett's Hospitals and Charities, 1904." 5s. net.**

**"Burdett's Official Nursing Directory." 8s. net.**

**"Cottage Hospitals, General, Fever, and Convalescent." 10s. 6d.**

**"Uniform System of Accounts for Hospitals and Public Institutions." New Edition. 4s. net.**

**"Hospital Expenditure: The Commissariat." 2s. 6d.**

**"A Legal Handbook for the Use of Hospital Authorities." 2s. 6d.**

**"The Cottage Hospital Case Book or Register of Patients." 10s. and 12s. 6d.**

**All these are published by the Scientific Press, Ltd., and may be obtained through any bookseller, or direct from the publishers, 28 and 29 Southampton Street, Strand, London, W.C.**

### JUST THE BOOK YOU WANT.

### HOW TO BECOME A NURSE.

THE NURSING PROFESSION: How and Where to Train. 2/- net, 2/4 post free.

By Sir HENRY BURDETT, K.C.B.

### HOW TO BECOME A NURSE.

THE NURSING PROFESSION 2/- net, 2/4 post free.

Contains a very complete list of Nurse-Training Schools in the United Kingdom and abroad.

### HOW TO BECOME A NURSE.

THE NURSING PROFESSION 2/- net, 2/4 post free.

Gives full details of Training, Premiums, Salaries, and Hours of Duty, &c., at each Institution.

### HOW TO BECOME A NURSE.

THE NURSING PROFESSION 2/- net, 2/4 post free.

Contains particulars of the Average Number of Yearly Vacancies in the various Institutions, together with full directions to whom to apply in each case.

*This Valuable Guide can be obtained from all Booksellers, or direct from the Publishers,*
THE SCIENTIFIC PRESS, LTD., 28 & 29 Southampton Street, Strand, London, W.C.

### EVERY NURSE SHOULD POSSESS

A COPY OF **THE NURSES' DICTIONARY.**

COMPILED BY

**HONNOR MORTEN.**

Price, in cloth, 2/- post free.
Bound in leather, 2/8 post free.

CONTAINING DESCRIPTIONS OF THE PRINCIPAL MEDICAL AND NURSING TERMS AND ABBREVIATIONS, INSTRUMENTS, DRUGS, DISEASES, ACCIDENTS, TREATMENTS, PHYSIOLOGICAL NAMES, OPERATIONS, FOODS, APPLIANCES, &c, &c., ENCOUNTERED IN THE WARD OR SICK-ROOM.

SUITABLE SIZE FOR THE APRON POCKET.

*Of all Booksellers, or of*

THE SCIENTIFIC PRESS, LTD., 28 & 29 Southampton Street, Strand, LONDON, W.C.

